# Comparative efficacy and safety of the minimally invasive ilioinguinal approach for anterior pelvic ring fracture: A protocol for systematic review and network meta-analysis

**DOI:** 10.1097/MD.0000000000028377

**Published:** 2021-12-30

**Authors:** Xiaohua Shi, Xiang Li, Aiguo Wang

**Affiliations:** Department of Minimally invasive Orthopaedics, Zhengzhou Orthopaedic Hospital, Zhengzhou, Henan, China.

**Keywords:** ilioinguinal approach, minimally invasive, network meta-analysis, pelvic fracture, protocol

## Abstract

**Background:**

Anterior pelvic ring contributes up to 40% of the stability of the pelvic ring and is located in close proximity to key pelvic organs, blood vessels, and nerves. An anterior pelvic ring fracture causes severe pain and is a potentially life-threatening condition in trauma patients. Currently available surgical repair methods are highly technical and include high risks of complications. The minimally invasive ilioinguinal approach (MIIA) is an emerging technique that reduces the risk of femoral nerve and external iliac vessel injury. However, the safety and efficacy of this technique have yet to be systematically scrutinized. This study outlines a proposed protocol for a network meta-analysis that investigates the efficacy of MIIA for anterior pelvic ring fracture.

**Methods:**

This study will utilize both Chinese and English language databases. All randomized controlled trials studying the use of MIIA for anterior pelvic ring fracture from January 2016 to May 2021 will be incorporated. Researchers will screen for literature that fits the inclusion criteria, followed by an assessment of risk bias and, finally, data extraction.

**Results:**

The Bayesian network meta-analysis will be used to evaluate all available Stata 14.0 and WinBUGS software.

**Conclusion:**

Our research aims to uncover the clinical utility of the MIIA approach for anterior pelvic ring fractures.

**Ethics and dissemination:**

Not required

**INPLASY registration number:**

INPLASY2021110020.

## 1 Introduction

Up to 3% of all fractures are pelvic fractures. These fractures often arise from high-energy injury.^[1]^ Surgery for pelvic fracture repair is challenging, given the complex anatomy and its diverse fracture morphology. The anterior pelvic ring is enclosed by the bladder, urethra, inguinal canal, blood vessels, and nerves. Pelvic fracture repair carries the risk of significant complications such as sexual dysfunction, malunion, and limb shortening, all of which carry high rates of patient morbidity.

At present, the most common fixation method for anterior pelvic ring fractures is open reduction and internal plate fixation, with the ilioinguinal approach being the most classic approach. However, this surgical technique is traumatic, complicated, and comes with a steep learning curve.^[2]^ The continuous improvement of the Stoppa approach simplifies the surgery but is offset by the additional difficulty of achieving adequate surgical exposure, especially in obese patients.^[3]^ External fixators are occasionally used to manage patients with open fractures, but the long-term use of an external fixator is often tolerated poorly by patients.^[4]^

The minimally invasive ilioinguinal approach (MIIA) negates the need to dissect the middle pelvic window, thus avoiding excessive tissue trauma and complications associated with the traditional ilioinguinal approach. One of the advantages of the MIIA is the reduced likelihood of femoral nerve and iliac vessel injury as well as the reduced risk of lower extremity deep venous thrombosis. MIIA is also able to achieve a satisfactory reduction of a pubic bone fracture without direct vision, which translates to reduced intraoperative blood loss and operating time. The MIIA technique also does not require dissection of the inguinal hernia, thus lowering the chances of a post-operative inguinal hernia. Moreover, peri-acetabulum screws are not used in this technique, which reduces the need for intraoperative fluoroscopy. Lastly, good plate bending prior to the fracture fixation further assists in shortening operative time and incidence of complications.

## 2 Materials and methods

### 2.1 Study registration

This study will be performed based on the PRISMA-P guidelines and using the Bayesian network meta-analysis. This study is registered on the International Platform of Registered Systematic Review and Meta-analysis Protocols (INPLASY), and the ID of the registered study is INPLASY2021110020 (https://inplasy.com/inplasy-2021-11-0020/).

### 2.2 Inclusion criteria

#### 2.2.1 Type of study

All relevant randomized controlled trials analyzing the MIIA approach for anterior pelvic ring fracture repair in English or Chinese will be compiled.

#### 2.2.2 Participants

All participants should be diagnosed with an anterior pelvic ring fracture based on current guidelines. There are no limitations in terms of gender, ethnicity, duration of injury, severity, or age.

#### 2.2.3 Interventions

All studies involved must include a control group that was managed with the ilioinguinal open reduction approach, while the intervention group must have received MIIA open reduction. The course of patient treatments that were unrelated to anterior pelvic fractures was disregarded from the analysis.

#### 2.2.4 Outcomes

The data we collected include operation time, intraoperative blood loss, “Matta Radiographic Score,” “Majeed Pelvic Score,” and complications.[^5^,^6^] Further details on these parameters are as follows:

- Operation time and intraoperative blood loss are used to evaluate the invasiveness and safety of surgery.
- The “Matta Radiographic Score” is used to evaluate whether the reduction of the fracture fulfills anatomical requirements (within 1 mm) or satisfactory reduction requirements (between 1–3 mm).
- The “Majeed Pelvic Score” is a 100-point score that comprises four grades: Excellent, above 85; good, 70 to 84; fair, 55 to 69; and poor, below 55. Some of the main parameters included in this score are pain and the impact of the injury on work, sitting, sexual function, and standing.
- The type and incidence of complications will also be recorded.
- Additional factors that impact the surgery and post-operative recovery will also be recorded.

All included literature must include at least one or more of the aforementioned parameters.

### 2.3 Database and search strategy

Papers published between January 2016 to May 2021 in popular databases such as the Wanfang database, Cochrane Library, China National Knowledge Infrastructure, EMBASE, China BioMedical Literature, PubMed, and Web of Science are to be included in this study. The search methods will involve medical subject headings and free-text terms, such as “Anterior Pelvic Ring,” “Fracture,” “Minimal Invasive, Ilioinguinal Approach,” “Safety,” and “Randomized controlled trial.” Table 1 depicts a comprehensive example of a search strategy that will be used in PubMed. Ongoing clinical trials registered on the International Clinical Trial Registration Platform will also be included.

**Table 1:** Detailed search strategy for PubMed.

NO.	Search item
1#	“pelvic, fracture”[MeSH Terms]
2#	“anterior pelvic ring fracture”[Title/Abstract]OR ((“pubic branch fracture”[Title/Abstract] OR “fracture of superior ramus of pubis”[Title/Abstrac] OR “fracture of inferior ramus of pubis”[Title/Abstrac] OR “fracture of superior and inferior ramus of pubis”[Title/Abstract] OR “pubic symphysis separation”[Title/Abstract]
3#	1# OR 2#
4#	“Surgical therapies”[MeSH Terms]
5#	“Open reduction and internal fixation”[Title/Abstract] OR “plate fixation”[Title/Abstract] OR “minimally invasive”[Title/Abstract] OR “ilioinguinal approach”[Title/Abstract] OR “minimally invasive ilioinguinal approach”[Title/Abstract]
6#	4# OR 5#
7#	“randomized controlled trial”[Title/Abstract] OR “controlled clinical trial”[Title/Abstract] OR “Randomized”[Title/Abstract] OR “random allocation”[Title/Abstract] OR “Randomly”[Title/Abstract]
8#	3# AND 6# AND 7#

### 2.4 Study selection and data extraction

Literature screening will be conducted independently by two researchers. Duplicate literature will be identified and excluded. All literature that fails to meet the established inclusion and exclusion criterion is also to be excluded. In cases where there is a discrepancy between the two researchers, a third researcher will be consulted to resolve the dispute. Basic information to be extracted from each study include clinicodemographic profiles, types of interventions, postoperative complications, and patient outcomes.

### 2.5 Risk of bias assessment

The quality of included studies is rated based on seven parameters as published in the Cochrane Handbook.^[7]^ For each parameter, a low risk of bias is defined as the correct use of the parameter, an unclear risk of bias being the unclear use of the parameter, and a high risk of bias is the incorrect or absence of a parameter. The risk of bias assessment for each included paper will be evaluated independently by two researchers. All cases of discrepancies between the two will be resolved by a third researcher.

### 2.6 Statistical analysis

The sampling method that will be used in this study is the Markov chain Monte Carlo method, which is often employed to solve random sampling simulation problems of distribution that are unable to be directly sampled. A Bayesian meta-analysis using the STATA14.0 software and Markov chain Monte Carlo method is used to extract data from included studies. Three Markov chains will be used for the simulation with 50,000 iterations used.

A reticular diagram that allows a clear depiction of various comparators will be created using STATA14.0. The RoR and its corresponding 95% confidence interval (CI) will also be calculated. A higher consistency level is one that has a lower limit of the 95% CI closer to 1. A fixed-effect model will be used in situations where the RoR is approximately 1. Otherwise, the random effect model will be incorporated. Dichotomous data will be expressed in terms of odds ratio and 95% CI (*P* < .05), while the WinBUGS1.4.3^[8]^ and area under the curve will be used to evaluate the efficacy of various interventions.

### 2.7 Assessment of heterogeneity

A fixed-effect model will be used if *P* > .10 and *I* ^*2*^ < 50%. In cases where the source of study heterogeneity is unable to be identified, a random-effect model will be used. A descriptive analysis will be used in cases where there is a large degree of clinical heterogeneity.

### 2.8 Subgroup analysis and sensitivity analysis

In the event that we obtain sufficient data, we plan to group multiple sensitivity analyses to assess the reliability and robustness of the combined results of a meta-analysis. Each study will then be excluded one by one as necessary before the overall effects are merged. Changes in heterogeneity that occur after article exclusion indicate that the excluded article is the source of heterogeneity.

### 2.9 Evaluation of publication bias

Inverted funnel diagrams will be used to determine the publication bias with regards to operation time, intraoperative blood loss, Matta Radiographic Score, Majeed Pelvic Score, and the incidence of complications.

### 2.10 Grading the quality of evidence

The Grading of Recommendations Assessment, Development and Evaluation framework will be used to appraise the quality of evidence extracted from the network and pairwise meta-analyses.[^9^,^10^]

## 3 Discussion

In 1960, the ilioinguinal approach was first introduced by Letoumel as a means of accessing the pelvic ring for managing pelvic fractures.^[11]^ This surgical approach provides a sufficient operative field of vision, which is from the pubic symphysis to the upper surface of the quadrilateral muscle, and then to the innominate bone plate in front of the sacroiliac joint. Nevertheless, the ilioinguinal approach also has obvious disadvantages, the first of these being the risk of significant surgical trauma given the need for an extensive dissection, a higher risk of blood vessel and nerve injury, as well as a higher risk of deep vein thrombosis.

Current reports find that the MIIA is able to achieve good results primarily due to there being no need to expose the middle to complete reduction, plate shaping, plate insertion, and fixation.^[12]^ This way, the MIIA method is able to reduce the risk of injury of the femoral nerve and external iliac vessels.

Minimally invasive surgery is a growing trend in the development of orthopedic surgery. Minimally invasive surgery involves not only a smaller skin incision but also a fundamental change in surgical methods and thinking. MIIA can preserve the integrity of the second window structure of the traditional ilioinguinal approach. In addition, the reduction of the broken end, the shaping of the steel plate, and the placement method are different. Less trauma and operation time also means less inflammation, less pain, and earlier return to pre-injury functionality. Based on this hypothesis, we seek to systematically compare the safety and efficacy of MIIA against the traditional network meta-analysis approach in order to formulate evidence-based recommendations and observations regarding the application of the MIIA technique in repairing anterior pelvic ring fractures.

## Author contributions

**Conceptualization:** Xiaohua Shi.

**Data curation:** Xiaohua Shi.

**Formal analysis:** Xiaohua Shi, Aiguo Wang.

**Funding acquisition:** Xiang Li.

**Investigation:** Xiaohua Shi.

**Methodology:** Xiaohua Shi, Aiguo Wang.

**Project administration:** Xiaohua Shi.

**Resources:** Xiaohua Shi.

**Software:** Xiaohua Shi, Aiguo Wang.

**Validation:** Xiaohua Shi.

**Visualization:** Xiaohua Shi.

**Writing – original draft:** Xiaohua Shi.

**Writing – review & editing:** Xiaohua Shi.
